# Benchmarking speech-to-text robustness in noisy emergency medical dialogues: an evaluation of models under realistic acoustic conditions

**DOI:** 10.1093/jamiaopen/ooaf147

**Published:** 2025-11-19

**Authors:** Denis Moser, Nikola Stanic, Murat Sariyar

**Affiliations:** School of Engineering and Computer Science, Bern University of Applied Sciences, Bern, Switzerland; School of Engineering and Computer Science, Bern University of Applied Sciences, Bern, Switzerland; School of Engineering and Computer Science, Bern University of Applied Sciences, Bern, Switzerland

**Keywords:** speech recognition, emergency medical services, speech-to-text, word error rate, clinical documentation

## Abstract

**Objectives:**

To evaluate the transcription accuracy of 6 German-capable speech-to-text (STT) systems in simulated emergency medical services (EMS) environments, focusing on clinically relevant performance under noisy and multilingual field conditions.

**Materials and Methods:**

We generated a corpus of 99 synthetic emergency dialogues and overlaid them with ecologically valid noise types—crowd chatter, traffic, public spaces, and ambulance interiors—at 5 signal-to-noise ratios (SNRs), producing 1980 noisy audio samples. Each was transcribed by 6 STT systems (recapp, Vosk, Whisper v3 variants, and RescueSpeech). We assessed performance using 5 metrics: Word Error Rate (WER), Medical Word Error Rate (mWER), TF–IDF Cosine Similarity, BLEU, and semantic embedding similarity. Statistical models quantified the effects of system, noise, and SNR on transcription fidelity.

**Results:**

recapp consistently outperformed all other systems across metrics. Among open-source models, Whisper v3 Turbo achieved the lowest mWER and strongest phrase-level accuracy (BLEU), while Whisper v3 Large preserved semantic content best. RescueSpeech and Vosk underperformed. “Inside crowded” noise had the most degrading impact on performance, while “talking” noise had minimal effect. Performance degradation was most pronounced at the lowest SNR (–2 dB).

**Discussion:**

STT model accuracy is highly sensitive to acoustic conditions. Clinically salient transcription errors (mWER) were most frequent under dense environmental noise. Whisper v3 Turbo balances accuracy and efficiency, suggesting strong potential for EMS applications.

**Conclusion:**

This study introduces a clinically grounded, noise-robust benchmark for STT evaluation in EMS settings. It highlights the importance of domain-specific metrics and acoustic realism for deploying STT systems where transcription errors carry safety-critical consequences.

## Introduction

In emergency medical services (EMS), language is often the first and most critical intervention.^1^ Upon arrival at the scene of a cardiac arrest, stroke, or trauma, paramedics rely on verbal communication to convey assessments, coordinate actions, and document life-saving decisions.^2^ Spoken reports, triage dialogues, and symptom descriptions form the foundation for downstream care, frequently preceding the entry of any information into electronic health records (EHRs). Yet this frontline speech is rarely documented in real time, instead depending on manual note-taking or delayed digital entry, processes that can introduce errors, increase clinician workload, and limit timely data availability.^3^

To modernize EMS workflows, speech-to-text (STT) technologies are increasingly proposed as a solution.^4^ By transcribing spoken input in real time, STT enables hands-free documentation, direct integration with EHRs, and the potential for AI-assisted decision support.^5^ Automating parts of the communication pipeline may reduce administrative burden while improving the continuity of care between prehospital and in-hospital settings.^6^

However, EMS presents far greater challenges for STT than controlled clinical environments.^7^ Field interactions occur in acoustically adverse conditions: crowded streets, echoing stairwells, siren-filled ambulances, or chaotic household settings.^8^ Speech may be interrupted, masked, or distorted by background noise. In multilingual regions such as Switzerland, additional complexity arises from dialectal variation, accents, and mixed-language utterances.^9^ For STT to be viable in such contexts, it must perform robustly not only in optimal scenarios but also under unpredictable acoustic and linguistic variability.^10^

Existing STT benchmarks do not adequately address these conditions.^5^^,^^11^^,^^12^ Most rely on curated corpora with clean audio, generic dialogues, or studio-quality recordings.^13^ Even in domain-specific evaluations, realistic noise profiles are rarely included.^14^ Furthermore, widely reported metrics such as word error rate (WER) treat all transcription errors equally, failing to distinguish between those with negligible impact and those with safety-critical consequences, such as misrecognizing a medication name or a procedural instruction. This disconnect between benchmark design and operational demands limits the ability of clinicians, developers, and policymakers to select or trust STT systems for EMS deployment.^15^

The implications of clinically relevant errors are clear. A reference transcript reading “Administer 1 mg adrenaline intravenously” could be misrecognized as “Administer 1 mg adrenaline intramuscularly,” altering the administration route and potentially endangering the patient. Similarly, “Salbutamol inhalation” might be transcribed as “Salicylate inhalation,” leading to inappropriate medication delivery. These examples highlight the need for evaluation methods that capture domain-specific accuracy, motivating the inclusion of a Medical Word Error Rate (mWER) tailored to EMS-critical terminology.

This study aims to evaluate the performance of modern STT systems under conditions that approximate the acoustic and linguistic challenges of German-speaking EMS. We assess transcription quality across a range of simulated emergency situations, encompassing indoor and outdoor locations and multiple noise environments representative of field operations. Our evaluation goes beyond conventional WER by adopting a multi-dimensional framework comprising: (1) a Medical Word Error Rate (mWER) to measure the accurate recognition of diagnoses, procedures, and drug names; (2) lexical similarity metrics (TF–IDF cosine similarity and BLEU) to assess phrase- and word-level fidelity; and (3) semantic similarity measures to evaluate meaning preservation despite lexical variation.

This work contributes to the field in 4 ways. First, it provides a replicable benchmarking framework that reflects the operational realities of prehospital care. Second, it introduces a clinically grounded metric (mWER) to support interpretability for healthcare practitioners and informaticians. Third, it offers comparative performance data across a range of STT architectures, including recent transformer-based systems and a domain-adapted rescue-dialogue model. Finally, it identifies specific noise environments—such as low signal-to-noise ratio (SNR) “inside crowded” conditions—where even top-performing models exhibit marked degradation, highlighting areas where further robustness improvements are needed.

In the sections that follow, we describe our data generation pipeline, model selection criteria, noise simulation methods, and evaluation metrics. We then present a comparative analysis of model performance under varied acoustic conditions, followed by a discussion of implications for EMS practice, system design, and future evaluation standards.

## Methods

To rigorously assess the suitability of STT systems for real-world EMS, we evaluated 6 models under acoustic conditions that realistically simulate German-speaking out-of-hospital and in-transport rescue scenarios. Since no public dataset captures the full range of noise constellations relevant to EMS practice, we developed a controlled and reproducible evaluation framework by generating synthetic emergency dialogues and overlaying them with representative background noises at varying intensities. Each model’s output was then compared to ground-truth transcripts using lexical and semantic metrics, with statistical analyses performed to quantify performance differences.

### Dataset generation

#### Bare-bone dialogues

To establish a controlled STT evaluation testbed, we used 99 synthetic MIMIC-IV–based transcripts (MIMIC-IV being a large, de-identified critical care database) generated in a prior study for structured German-language EMS use cases.^16^ These were rendered into speech using the open-source Piper TTS tool,^17^ which provides native German speaker profiles. To introduce realistic challenges, we varied synthetic voices and medical scenarios, producing 99 WAV files with diverse prosody and speaker turn-taking.

#### Noise overlay

To simulate the acoustic conditions typically encountered by EMS personnel, each synthetic dialogue was overlaid with 1 of 4 ecologically valid background noise types. These were sourced from publicly licensed audio libraries: BBC Rewind^18^ (datasets 07026103 and 07060050 for the first 2 noise types) and ZapSplat^19^ (using terminal ambience and interior ambulance recordings for the latter 2). The selected noise profiles reflect common EMS deployment settings:

- **Talking**: Indoor crowd chatter, such as restaurant or household conversations;
- **Outside traffic**: Roadside and highway traffic noise;
- **Inside crowded**: Sounds from large enclosed public spaces, eg, train stations;
- **Inside ambulance**: Interior ambulance noise, including engine, road vibration, and sirens.

The selection is grounded in empirical dispatch statistics,^20^^,^^21^ which show that approximately 70-75% of Swiss out-of-hospital emergencies occur in private residences, with the remainder in public or mobile contexts. Accordingly, these 4 categories model key operational settings for German-speaking rescue workers.

To systematically evaluate model robustness under realistic acoustic stress, we overlaid each of the 99 clean speech recordings with all 4 background noise types at 5 distinct intensity levels: −15, −20, −25, −30, and −35 dBFS. These levels correspond approximately to RMS signal-to-noise ratios (SNRs) ranging from −2 dB (highly degraded conditions where noise exceeds the signal) to +18 dB (near-optimal intelligibility). This SNR spectrum aligns with thresholds commonly cited in auditory perception and ASR research, where 20 dB is considered the upper limit of reliable intelligibility in noisy environments.^22–24^ The resulting dataset comprised 1980 noisy audio files (99 dialogues × 4 noise conditions × 5 noise levels), each paired with its original reference transcript (average length ∼1670 words). This design enables a controlled yet ecologically valid testbed that reflects the real-world variability EMS personnel face, while preserving analytical rigor and reproducibility (comparable to Goel *et al*^25^).

### Speech-to-text models

To evaluate the practical suitability of STT systems for EMS, we selected 6 models covering commercial-grade tools, offline-capable engines, and recent transformer-based architectures. Selection criteria included support for German language transcription, adaptability to clinical or real-time contexts, and relevance to EMS documentation needs. Nuance/Dragon Medical was excluded due to its dictation-focused interfaces, licensing constraints, and lack of flexible file processing or API-first integration.^26^ Cloud-based medical STT services (such as Google Speech-to-Text Clinical Conversation and Amazon Transcribe Medical) were also excluded for lacking specialized German models, requiring cloud-only deployment, and being optimized for stationary clinical dictation rather than high-noise, multi-speaker EMS scenarios.

- **recapp: **A commercial Swiss STT platform designed for clinical environments and tuned to national dialects.
- **Vosk DE 0.21: **An open-source German model optimized for offline and low-latency transcription in telephony or edge deployments.
- **Whisper v3 Large: **OpenAI’s 1.55-billion-parameter multilingual model with strong performance across diverse languages.^27^
- **Whisper v3 Medium: **A 769-million-parameter variant optimized for faster inference with moderate accuracy trade-offs.
- **Whisper v3 Turbo: **A compact, high-speed variant (∼809 million parameters) tailored for real-time transcription with minimal degradation.
- **RescueSpeech: **A Whisper v2–based model fine-tuned on ∼1.6 hours of domain-specific German rescue dialogues.^28^

The commercial recapp system was accessed through its REST-API in March 2025. As the API evolves continuously, versioning is dynamic rather than fixed. recapp is based on the Whisper v2 architecture and fine-tuned on proprietary, high-quality data encompassing several thousand hours of Swiss German (gsw-CH) and High German speech, including extensive dialectal coverage. Technical details of further aspects of the system are documented in its patent CH717305A1. None of the authors are employed by or receive financial compensation from the developers of recapp. All evaluations were conducted independently using publicly available API endpoints.

The RescueSpeech German corpus comprises approximately 2 hours of speech material, corresponding to about 1 hour 36 minutes (≈1.6 hours) of annotated, clean data. The actual training duration varies by sub-task and fine-tuning strategy. (1) Acoustic-model (ASR) fine-tuning: the “clean” training split used for ASR adaptation contains roughly 61.86 minutes (just over 1 hour) of speech. (2) Speech-enhancement/multi-condition training: for robustness tasks, a “noisy” augmented version of the dataset was generated using synthetic noise, producing about 7.2 hours of material.

All 1980 synthetic emergency recordings were transcribed by each model, yielding 11 880 output transcripts for comparative analysis under controlled noise conditions.

### Evaluation

To evaluate transcription performance across diverse acoustic conditions, we applied a multi-layered strategy that combines lexical accuracy, domain-specific sensitivity, and semantic preservation. Our evaluation proceeds in 3 stages:

- **Metric calculation**: We apply 5 complementary metrics—Word Error Rate (WER), Medical Word Error Rate (mWER), TF–IDF Cosine Similarity, BLEU score, and semantic embedding similarity—to capture different dimensions of STT output quality.
- **Preprocessing and normalization**: Given the variability in STT output formats (eg, casing, punctuation, abbreviation use), we implement a consistent normalization pipeline to ensure comparability across systems.
- **Statistical modeling**: We use regression and machine learning models to quantify the effects of STT system, noise type, and noise intensity on transcription performance, with robust methods to account for non-independence across repeated measures.

This layered evaluation design ensures that we not only quantify raw accuracy but also assess clinically relevant fidelity and robustness under realistic EMS noise conditions.

### Implementation of evaluation metrics

We implemented all metrics in Python and applied them to the 11 880 STT outputs. Because STT systems differed in formatting (eg, RescueSpeech outputs uppercase without punctuation, Vosk uses lowercase only), we applied a uniform preprocessing step: lowercasing, punctuation removal, whitespace normalization, and abbreviation expansion. This standardization enabled fair, model-agnostic comparisons.

#### Word error rate (WER)

WER quantifies the proportion of word-level substitutions, deletions, and insertions between the reference and hypothesis transcript. We computed WER using the jiwer library, following a shared preprocessing pipeline applied to both reference and hypothesis texts. German-specific features (eg, “Dr” → “Doktor”) were normalized inline, and transcripts were tokenized into word lists suitable for WER calculation on a per-document basis.

#### Medical word error rate (mWER)

To capture clinical relevance, we extended the WER metric to target medical terminology. The mWER isolates transcription errors affecting medically salient terms (diagnoses, procedures, and drugs), which are critical for EMS documentation and downstream decision-making.

We reused jiwer’s alignment logic but added a second layer of classification. Substitutions, insertions, and deletions were tagged as “medical” if they involved terms matching a curated German medical vocabulary. Words were embedded using a character-level TF–IDF vectorizer, and phrases were marked as medical errors when any constituent word showed cosine similarity > 0.80 with the lookup dictionary.

The lookup vocabulary was assembled from 3 structured sources:

- **Diagnoses**: ICD codes from MIMIC-IV were mapped to UMLS CUIs using the MRCONSO metathesaurus,^29^ and German synonyms were extracted, normalized, and filtered.
- **Procedures**: The German LOINC translation (deDE15) from BfArM^30^ provided standardized procedure names.
- **Drugs**: The 2025 ATC classification (WidO^31^) yielded German drug names, deduplicated and normalized.

These resources were consolidated into CSV dictionaries used for mWER tagging, enabling a direct measure of how well each model preserves medical information in noisy environments.

The mWER metric allows us to focus on the subset of linguistic information that directly affects structured EMS documentation and downstream protocol filling. While colloquial and situational expressions (eg, collapsed, no pulse, short of breath) are integral to real-world communication, their inclusion in the error count depends strongly on the intended application. For systems designed to automatically complete formalized medical reports, excluding these terms is advantageous, as mWER isolates clinically codifiable information. Conversely, in applications aiming to support live triage assistance or situational awareness, incorporating such colloquial descriptors could be beneficial. Thus, the optimal definition of mWER is use-case dependent, and this work prioritizes concepts relevant for structured EMS protocol generation.

#### Lexical similarity metrics

Lexical similarity metrics provide insight into word choice and phrasing beyond raw error rates. They are particularly useful when evaluating models that may paraphrase or restructure output while remaining intelligible or accurate.

- **TF–IDF Cosine Similarity**: We vectorized transcripts using scikit-learn’s TfidfVectorizer, removing German stopwords via spaCy and applying consistent preprocessing. Cosine similarity was then computed between each hypothesis–reference pair to yield a per-document lexical overlap score.
- **BLEU**: To measure n-gram overlap, we used sacreBLEU with the “13a” tokenizer. Only lowercasing was applied. BLEU is sensitive to exact matches, helping to reveal degradation in phrase structure under noisy conditions.

#### Semantic similarity

To assess whether STT outputs convey the same meaning as reference transcripts despite lexical variation, we used semantic embedding similarity. Transcripts were normalized (lowercased, alphanumeric only, whitespace collapsed), then passed to OpenAI’s text-embedding-3-large model to obtain 3072-dimensional embeddings. Cosine similarity between each reference–hypothesis pair provided a document-level semantic match score.

### Statistical analysis

#### Core statistical evaluation

To quantify the influence of model, noise type, and noise level, we conducted statistical modeling on all 11 880 transcription instances. For each of the 5 metrics (WER, mWER, TF–IDF cosine similarity, BLEU, semantic embedding similarity), we computed:

- **Descriptive statistics**: Mean, median, standard deviation, min, and max by STT model and noise type.
- **Inferential models**: We fitted ordinary least squares (OLS) regressions using Python’s statsmodels library. Each metric served as the dependent variable; categorical predictors were STT system (baseline: recapp), noise type (baseline: outside traffic), and SNR level (baseline: –35 dBFS).

We validated assumptions via variance inflation factors (all VIFs < 5, see Table S1), residual-vs-fitted scatterplots, and Q–Q plots. Because assumptions of normality and homoscedasticity were violated, we applied cluster-robust standard errors, clustering on dialogue ID to account for repeated measures across conditions.

#### Confirmatory modeling

To validate our mWER findings, we performed 2 additional analyses:

- **Gradient Boosted Mixed Model**: Using the gpboost library, we fitted a tree-based model with group-level intercepts on dialogue ID, using an 80:20 holdout split and cross-validation for tuning. Feature importances were extracted via SHAP values.
- **Linear Mixed Effects Model**: We also fitted a model with fixed effects for system, noise type, and SNR, and random intercepts for dialogue ID using statsmodels. MixedLM.

These confirmatory models were conducted to evaluate the robustness of our primary findings—either to reinforce confidence in their validity or to reveal inconsistencies that might indicate areas requiring further investigation.

## Results

### Overview

**Figure 1.**
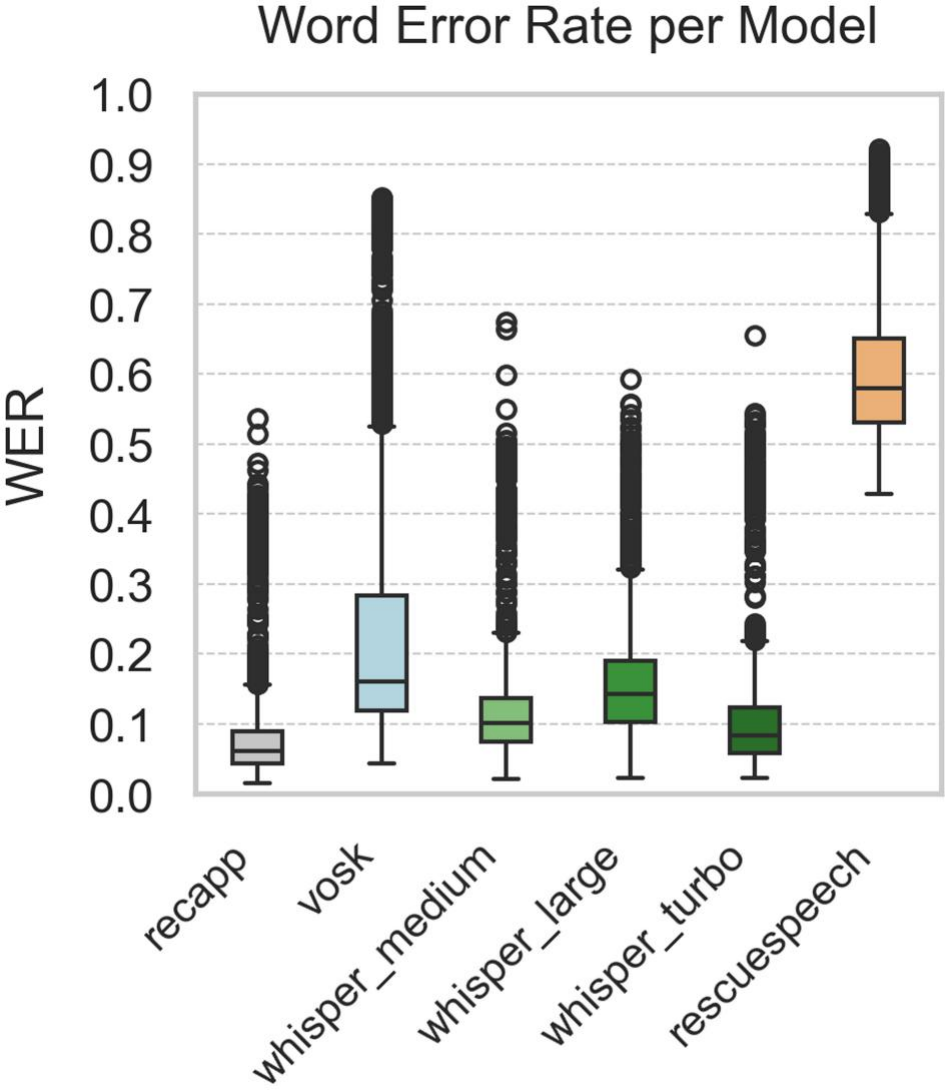
Boxplot of WER for each STT system (*n* = 1980 samples per model; total *n* = 11 880), calculated over 99 reference utterances × 20 noise conditions.

**Figure 2.**
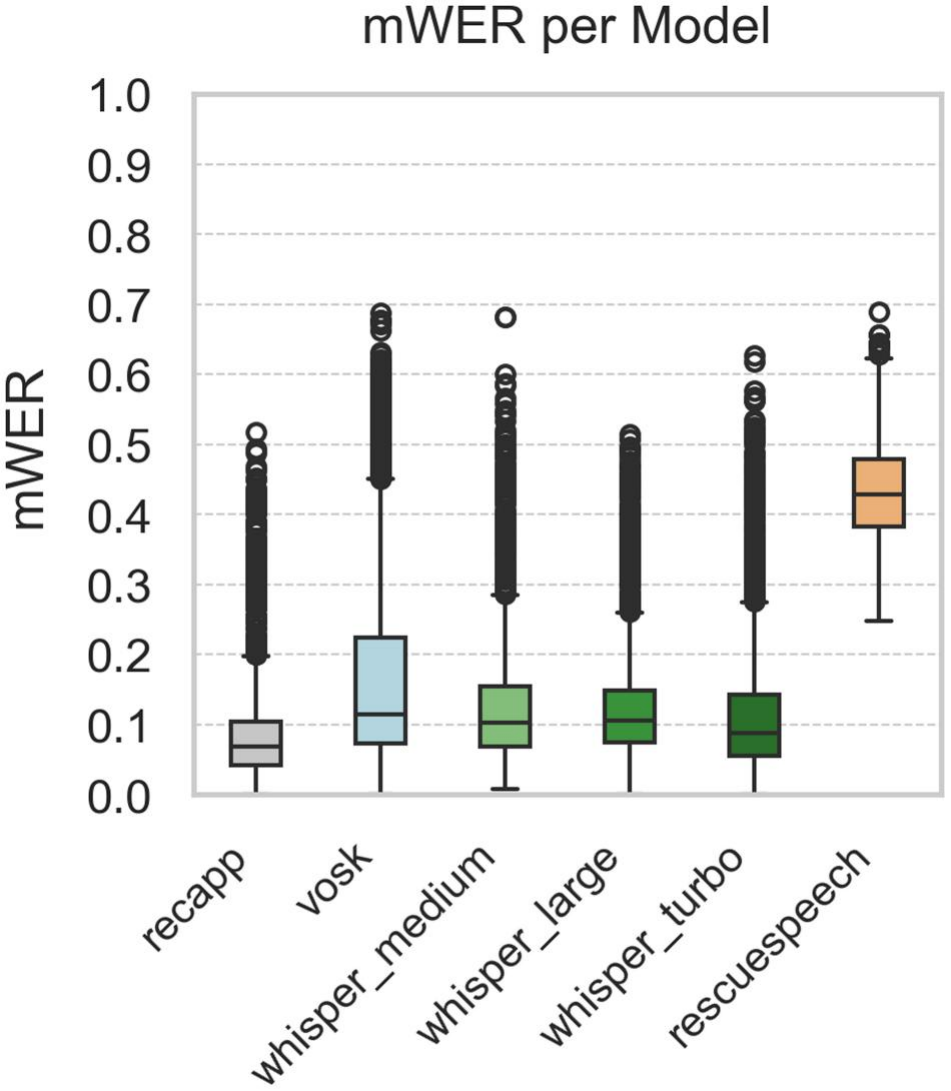
Boxplot of mWER for each STT system (*n* = 1980 samples per model; total *n* = 11 880), calculated over 99 reference utterances × 20 noise conditions.

**Figure 3.**
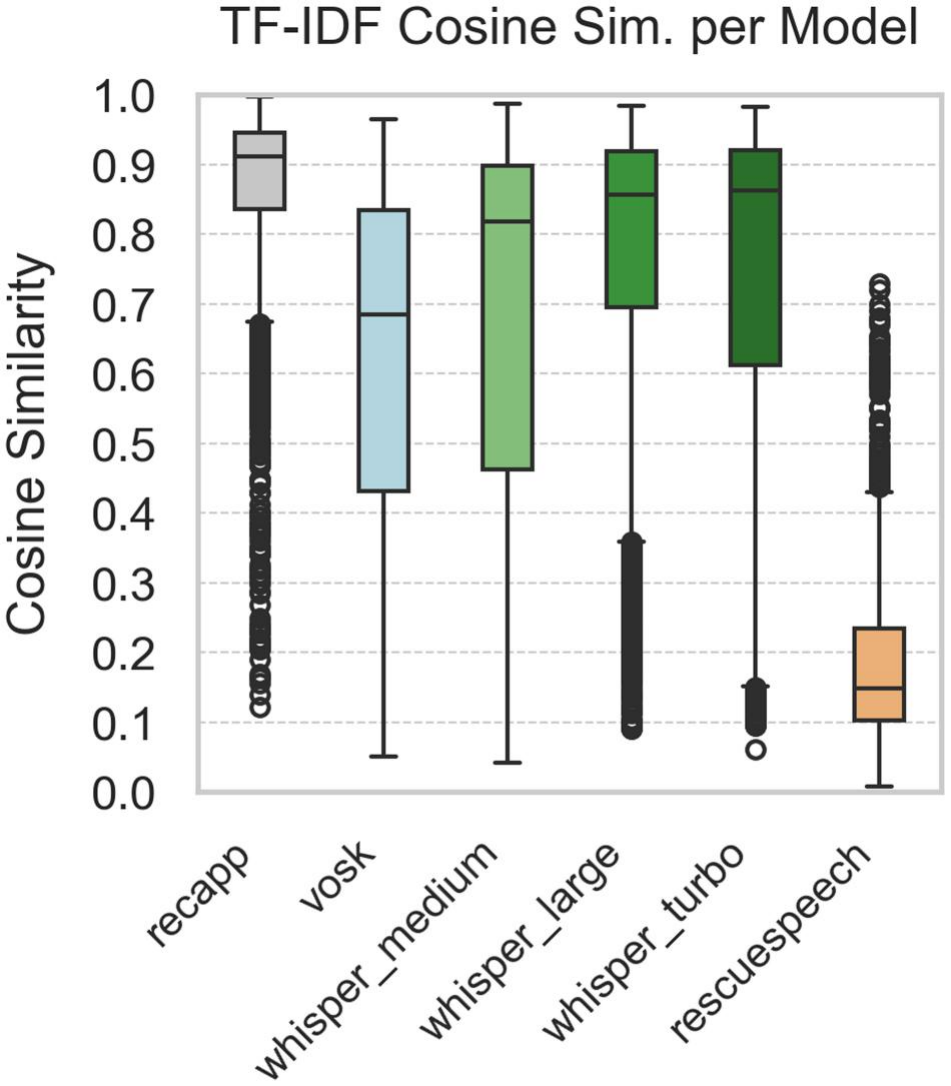
Boxplot of TF-IDF Cosine Similarity for each STT system (*n* = 1980 samples per model; total *n* = 11 880), calculated over 99 reference utterances × 20 noise conditions.

**Figure 4.**
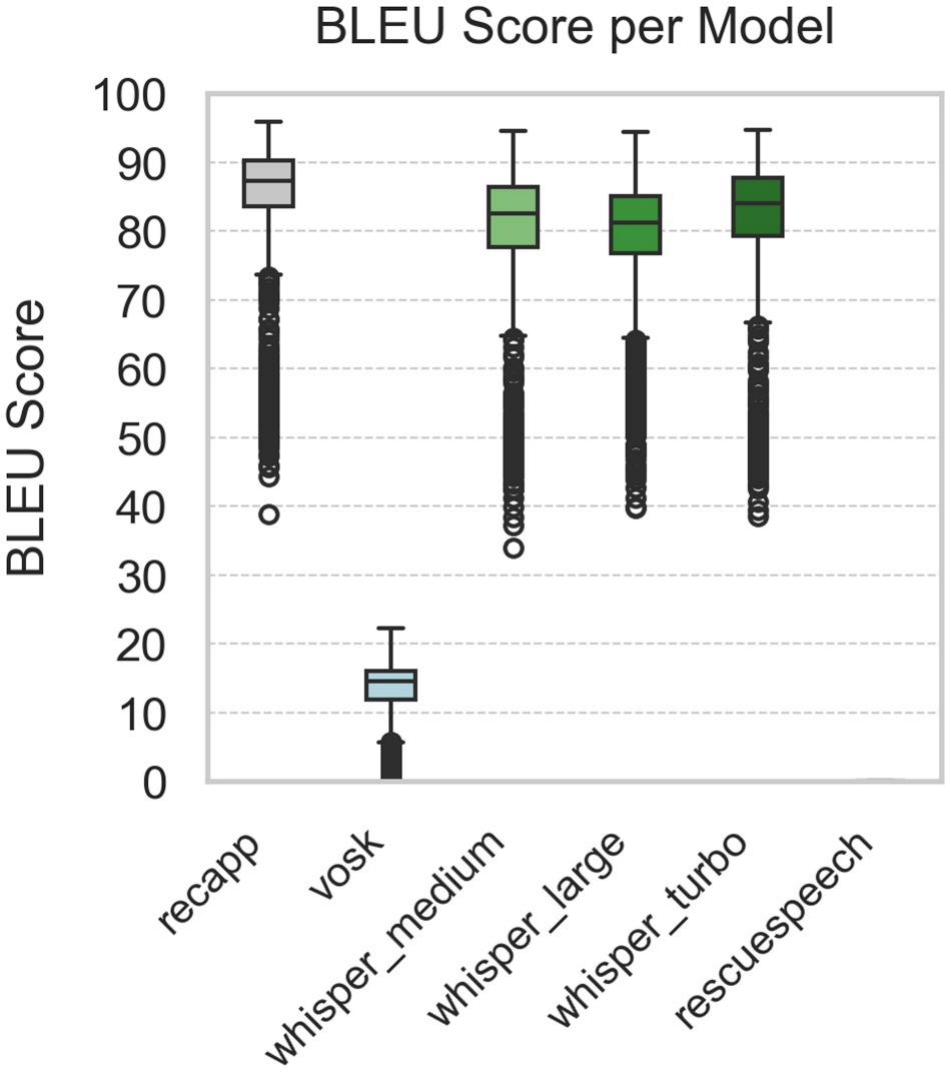
Boxplot of BLEU Score for each STT system (*n* = 1980 samples per model; total *n* = 11 880), calculated over 99 reference utterances × 20 noise conditions. The RescueSpeech model achieves BLEU scores near zero and therefore its box is not visible at this scale.

**Figure 5.**
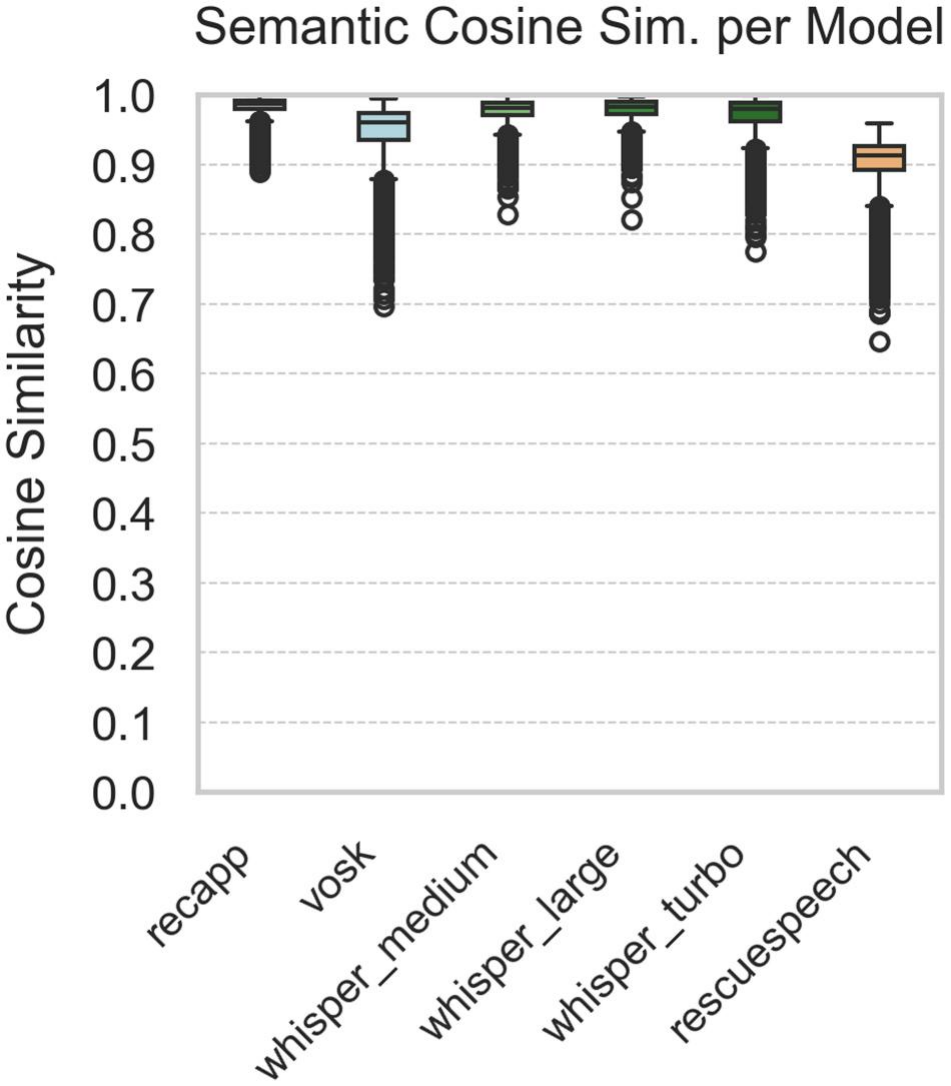
Boxplot of Semantic Cosine Similarity for each STT system (*n* = 1980 samples per model; total *n* = 11 880), calculated over 99 reference utterances × 20 noise conditions.

Figures 1-5 summarize the STT performance across 5 evaluation metrics, based on 1980 samples per model (99 dialogues × 20 noise conditions; 11 880 total comparisons). Figure 1 shows the WER, while Figure 2 reports the mWER. Figure 3 presents TF–IDF Cosine Similarity, and Figure 4 shows BLEU scores (note: RescueSpeech is not displayed due to near-zero values). Figure 5 displays Semantic Similarity, measured via cosine embedding distance, which reflects the preservation of meaning under noisy conditions. Boxplots disaggregated by noise profile and intensity level are provided in Figures S1-S5, while detailed descriptive statistics and OLS regression outputs can be found in Tables S1-S15.

### Descriptive performance by model

Overall, *recapp* outperformed all other STT systems across all metrics, achieving the lowest WER and mWER (Figures 1 and 2), highest lexical overlap (Figure 3), strongest BLEU scores (Figure 4), and excellent semantic similarity (Figure 5), with tight interquartile ranges indicating consistent performance. Among open-source models, *Whisper v3 Turbo* showed the best balance, with low WER and mWER and strong BLEU performance, making it well-suited for real-time EMS use. *Whisper v3 Large* led in TF–IDF and semantic similarity, indicating superior meaning preservation, though with slightly lower phrase-level accuracy.

In contrast, *RescueSpeech* underperformed across all dimensions, with near-zero BLEU scores and poor TF–IDF and semantic similarity, suggesting weak structural and clinical fidelity. *Vosk DE 0.21* achieved moderate error rates but showed substantial BLEU degradation, indicating limited ability to maintain phrasing under noise. These results highlight recapp’s robustness and the growing viability of open-source models like Whisper Turbo in EMS contexts.

### Model effects

Regression analysis confirmed that transcription performance varied significantly by STT model across all evaluation metrics (*P* < .05), with the exception of semantic similarity (*P* = .206). Table 1 summarizes the marginal effects of each model relative to the baseline (recapp), controlling for noise type and SNR.

**Table 1.** Comparison of STT models, noise types, and signal levels across WER, mWER, TF–IDF Cosine Similarity, BLEU Score, and Semantic Similarity. Lower WER/mWER and values closer to zero (or less negative) in similarity/score metrics indicate better performance.

Predictor	WER	mWER	TF–IDF Cosine similarity	BLEU score	Semantic similarity
Whisper V3 Large (Model)	0.075	0.035	−0.091	−5.514	−0.005
Whisper V3 Medium (Model)	0.039	0.040	−0.161	−4.791	−0.007
Whisper V3 Turbo (Model)	0.026	0.026	−0.116	−3.456	−0.014
Vosk DE 0.21 (Model)	0.156	0.083	−0.230	−71.818	−0.037
RescueSpeech (Model)	0.518	0.344	−0.676	−85.273	−0.080
Inside Ambulance (Noise Type)	0.019	0.019	−0.030	−1.016	−0.006
Inside Crowded (Noise Type)	0.079	0.067	−0.085	−5.134	−0.021
Talking (Noise Type)	0.007	0.009	−0.022	−0.408	−0.003
−15 dBFS (SNR: –2 dB)	0.196	0.167	−0.178	−11.471	−0.041
−20 dBFS (SNR: 3 dB)	0.062	0.057	−0.066	−3.171	−0.011
−25 dBFS (SNR: 8 dB)	0.018	0.017	−0.024	−0.864	−0.003
−30 dBFS (SNR: 13 dB)	0.004	0.004	−0.007	−0.120	−0.000

Color coding: green = better performance for that column, red = worse performance for that column. To improve readability, we present Table 1 with a visual separation between the 3 predictor categories (STT model, noise type, and SNR level) using bold horizontal lines. All coefficients represent marginal effects relative to the baseline system or reference (recapp). Absolute descriptive scores for recapp are provided in the supplementary material.

*RescueSpeech* exhibited the most substantial performance degradation, with WER increasing by 0.518, mWER by 0.344, and BLEU score dropping sharply by −85.273 points. It also showed the largest declines in TF–IDF similarity (−0.676) and semantic similarity (−0.080), indicating both lexical and conceptual failures under acoustic stress.

*Vosk DE 0.21* also underperformed, with WER rising by 0.156 and mWER by 0.083, along with large reductions in BLEU (−71.818) and TF–IDF similarity (−0.230). While its semantic similarity decline was more modest (−0.037), this pattern suggests that Vosk captures general content but struggles with precise phrasing and term-level accuracy.

Among open-source transformer models, *Whisper v3 Turbo* achieved the closest performance to recapp, with minimal increases in WER (+0.026) and mWER (+0.026), a relatively small BLEU reduction (−3.456), and moderate lexical degradation (TF–IDF: −0.116). This confirms its suitability for real-time EMS documentation, balancing robustness and efficiency.

*Whisper v3 Medium* followed closely, with slightly higher WER (+0.039) and mWER (+0.040), and a BLEU loss of −4.791. However, its TF–IDF drop (−0.161) suggests more structural paraphrasing compared to Turbo.

*Whisper v3 Large* showed the best performance in preserving semantic content (−0.005) and lexical similarity (−0.091), but this came at the cost of greater phrase-level degradation (BLEU: −5.514), suggesting a trade-off between meaning retention and exact surface-form reproduction.

In summary, recapp consistently outperforms open-source alternatives across all metrics, while Whisper v3 Turbo offers the best balance of accuracy and efficiency among freely available models.

### Noise-type effects

Using outside traffic as the reference condition, the “inside crowded” noise type had the most detrimental effect on transcription quality, particularly on BLEU scores, which dropped by −5.134, indicating a significant loss in phrase-level coherence. This condition also led to notable increases in WER (+0.079) and mWER (+0.067), and a decline in TF–IDF similarity (−0.085) and semantic similarity (−0.021), suggesting that dense, reverberant public environments introduce both structural and conceptual distortion.

In contrast, the “talking” condition had minimal impact across all metrics, with changes remaining below one point: WER (+0.007), mWER (+0.009), TF–IDF similarity (−0.022), and semantic similarity (−0.003). The “inside ambulance” setting produced intermediate degradation: WER (+0.019), mWER (+0.019), BLEU (−1.016), and semantic similarity (−0.006), likely reflecting consistent but less spectrally dense interference from sirens, vibration, and engine noise.

Error patterns differed by noise type: in “inside crowded” conditions, clinically significant substitutions occurred (eg, intravenous → intranasal, oxygen mask → oxygen mass), whereas “talking” noise mostly produced minor lexical changes (eg, patient → the patient) with minimal clinical impact.

In short, temporally and spectrally dense public-space noise (eg, “inside crowded”) is more disruptive than background speech-like noise (“talking”), confirming the need for heightened robustness in public and transport scenarios.

### SNR effects

Using outside traffic as the reference, transcription performance remained stable at higher SNR levels (−30 dBFS and –25 dBFS; ∼13 and 8 dB RMS), with minimal metric changes (eg, WER + 0.004 to +0.018; BLEU drop < 1 point). At –20 dBFS (SNR ∼3 dB), moderate degradation emerged: WER rose by 0.062, mWER by 0.057, and BLEU fell by −3.171.

At the lowest SNR (−15 dBFS; −2 dB), where noise exceeds speech, all lexical metrics declined sharply: WER increased by 0.196, mWER by 0.167, TF–IDF fell by −0.178, and BLEU dropped by −11.471. Semantic similarity declined only slightly (−0.041), suggesting core meaning was better preserved than surface form. These results confirm prior ASR thresholds and highlight 3 dB SNR as a critical tipping point for EMS transcription quality.

Overall, performance remains stable until approximately 3 dB SNR, below which degradation accelerates sharply, particularly at −2 dB where noise energy exceeds speech.

### Confirmatory analyses (mWER)

The 2 confirmatory analyses to validate the robustness of the mWER results yielded consistent and converging evidence. The linear mixed-effects model produced effect estimates that closely matched those of the primary OLS model (see Table S16). The GPBoost model further confirmed the prominence of RescueSpeech, −15 dBFS noise, and inside crowded noise as the strongest contributors to mWER degradation, as visualized through SHAP-based feature attribution (see Figures S6 and S7). Together, these results reinforce the reliability of the observed system- and condition-level effects across distinct statistical approaches.

## Discussion

This study assessed the robustness of 6 STT systems under realistic noise conditions encountered in German EMS. Performance varied substantially, yielding practical guidance for selecting models suited to real-time documentation and clinical decision support in noisy, high-stakes settings.

The commercial model recapp consistently outperformed all other systems across lexical and semantic metrics. Among open-source models, Whisper v3 Turbo emerged as the most balanced performer, achieving the lowest mWER and best BLEU scores, while Whisper v3 Large showed marginally stronger results in semantic similarity and lexical overlap. Notably, Turbo’s design—pruning Large’s decoder layers from 32 to 4—maintained high transcription quality while enabling faster inference, making it especially attractive for latency-sensitive EMS applications. In contrast, the RescueSpeech and Vosk models underperformed, with RescueSpeech showing degraded accuracy despite domain-specific fine-tuning, likely due to its limited training corpus and older architecture.

RescueSpeech’s weaker performance—despite being Whisper-based—stems primarily from the small and homogeneous fine-tuning set. With <2 hours of clean, annotated audio, the model simply has not seen the acoustic and linguistic variability typical of emergency calls (background noise, channel shifts, accents, dialect mixing, code-switching). Noise augmentation may stretch the signal to ∼7 hours, but it does not create new lexical, dialectal, or prosodic diversity, so generalization remains limited. By contrast, recapp (also Whisper-based) is fine-tuned on thousands of hours of high-quality Swiss German and High German speech spanning multiple dialects and accents, which predictably yields more robust transcription in EMS contexts. In short, dataset scale and diversity dominate performance here, echoing the long-standing result that data often matters more than the specific model architecture.^32^

One of the more unexpected findings was the minimal degradation caused by the “talking” noise condition. While this category introduced sporadic background speech, it seems to interfere less with STT models than denser, more uniform environmental sounds. In contrast, “inside crowded” noise—representing public spaces like train stations—had the strongest negative impact on phrase-level coherence and medical term recognition. This suggests that the temporal and spectral density of ambient sound may be more disruptive than the mere presence of speech-like noise. These patterns support the notion that public and mobile EMS settings require heightened model resilience, while residential scenarios present comparatively favorable acoustic conditions.

Despite these contributions, several limitations must be acknowledged. First, the synthetic nature of the test set—constructed via text-to-speech (TTS) synthesis—may overestimate difficulty in some cases. TTS-generated speech lacks natural prosodic variability and hesitation phenomena, which could influence error rates, especially in models that have been trained on naturalistic data. While this synthetic setup allowed precise control over linguistic content and noise overlays, factors present in real-world recordings—such as variable microphone quality, and patient conditions like distress, pain, or impaired speech^33^—may lead to higher WER and mWER values.^34^

Second, our medical error metric (mWER) is lexicon-based and was constrained to diagnoses, procedures, and medications drawn from MIMIC-IV and related sources. While this provides a structured, reproducible way to quantify domain-specific errors, it may underrepresent clinically important terms outside these categories, such as symptoms, actions, or colloquial descriptors used in field communication. As such, mWER reflects only a subset of the vocabulary critical to EMS documentation.

Third, temporal alignment might important performance dimensions for speech-to-text systems. Their relevance, however, varies with the use case. The study focused on automatic completion of structured EMS documentation from monologic or short-turn speech, where diarization plays a secondary role. In contrast, diarization accuracy is critical in contexts such as team communication analysis, multi-speaker debriefings, and call-center triage, where correct speaker attribution is essential. For our protocol-filling objective, transcription accuracy outweighs diarization performance. In parallel research, we employ open source diarization systems fine-tuned on manually annotated corpora featuring rapid speaker changes; nevertheless, these experiments fall outside the present paper’s scope.

Looking forward, future work should incorporate still very rare real-world EMS audio recordings—captured with informed consent and de-identified—into model evaluation pipelines. This would allow validation under authentic prosodic and environmental conditions beyond simulations.^35^ Expanding the medical vocabulary to include symptom terms, action verbs, and abbreviations frequently used by paramedics could also improve the sensitivity of mWER-like metrics. Finally, STT evaluation should move beyond plain text accuracy to assess structured output extraction, such as speaker turns, timestamps, or punctuation—elements essential for seamless integration into EMS reporting systems and electronic health records.

## Conclusion

Our study demonstrates that STT model performance in EMS-like environments depends not only on model architecture but also on noise type, intensity, and the specific evaluation metric. While proprietary systems such as recapp offer consistently strong performance, open-source alternatives, particularly Whisper v3 Turbo, can achieve near-parity with the added benefits of speed and transparency. Commercial systems may also be adapted to specific requirements through provider-led customization, whereas similar adaptations for open-source models require in-house expertise and resources. These findings offer practical guidance for deploying STT systems in the field and provide a reproducible benchmark for ongoing model evaluation in noisy, high-stakes clinical settings.

## Supplementary Material

ooaf147_Supplementary_Data

## Data Availability

All evaluated models are publicly accessible: Whisper v3 (Large, Medium, Turbo) via https://github.com/openai/whisper; Vosk DE 0.21 from https://alphacephei.com/vosk; and RescueSpeech (German) from https://github.com/rescuespeech-DE. The commercial recapp system was accessed via its REST-API (March 2025) under a research agreement. The benchmark dataset contains synthetic German emergency-audio recordings and corresponding ground-truth resources for STT robustness testing: 1980 WAV files from 99 dialogue texts mixed with 4 noise types at 5 loudness levels, plus a unified MongoDB collection (∼11 880 entries) linking all STT outputs and references via identifiers (eg, convoID, ambientVariant, processedVolume). All data are fully synthetic and non-sensitive, available at https://doi.org/10.5061/dryad.gtht76j1b. Further supplementary materials—including dataset-generation details, evaluation scripts, and API endpoint instructions—are provided at https://github.com/denMo24/stt-emergency-benchmark.
