# Supplementary material for: Benchmarking speech-to-text robustness in noisy emergency medical dialogues: an evaluation of models under realistic acoustic conditions
Source: JAMIA Open. 2025 Nov 19;8(6):ooaf147. doi: 10.1093/jamiaopen/ooaf147 (PMC12628192; doi:10.1093/jamiaopen/ooaf147)

Supplementary Figures

Figure S1. Boxplots of WER for six STT models across four noise types (rows) and five noise volume levels (columns); n = 99 per box (total n = 11 880).


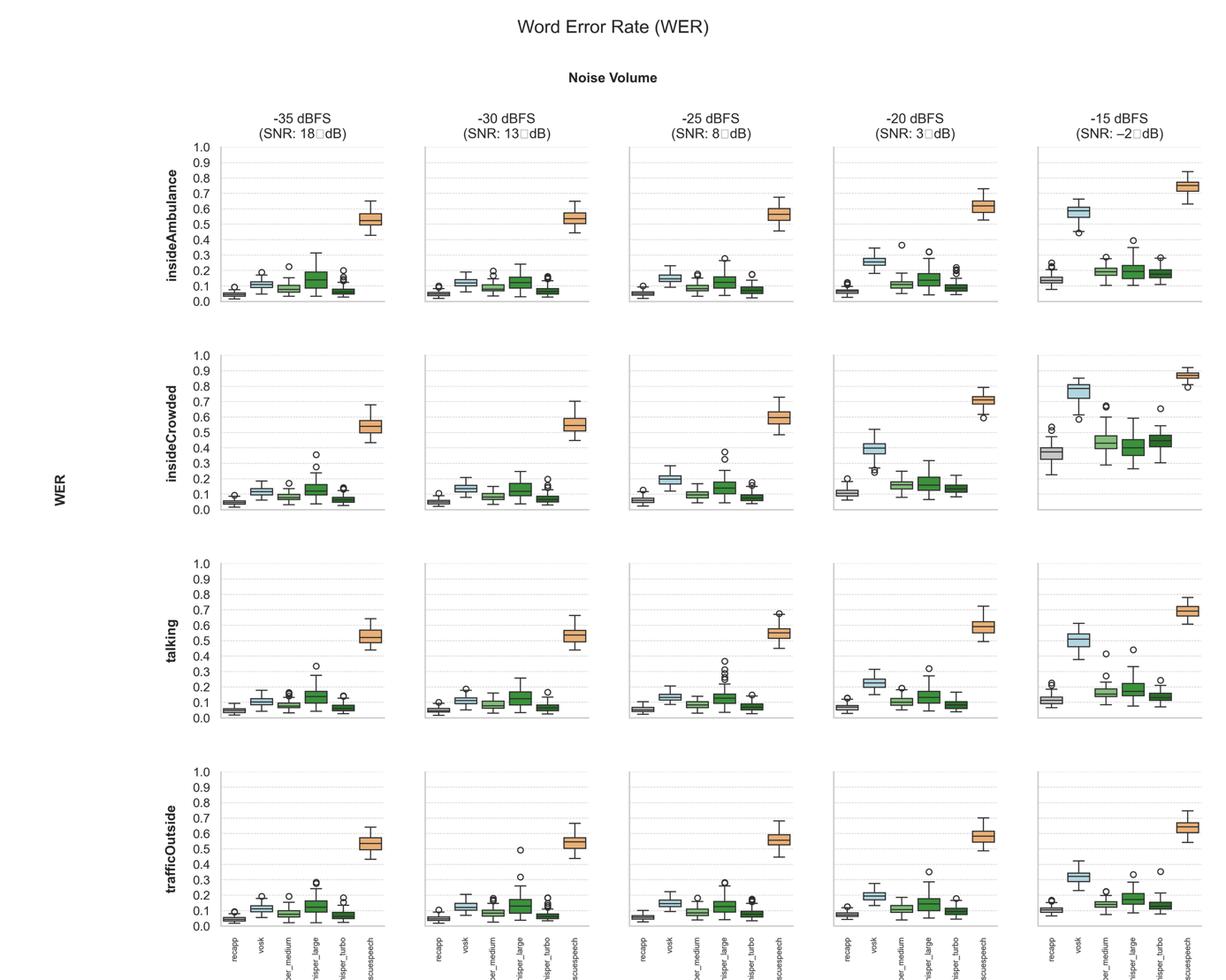


Figure S2. Boxplots of mWER for six STT models across four noise types (rows) and five noise volume levels (columns); n = 99 per box (total n = 11 880).


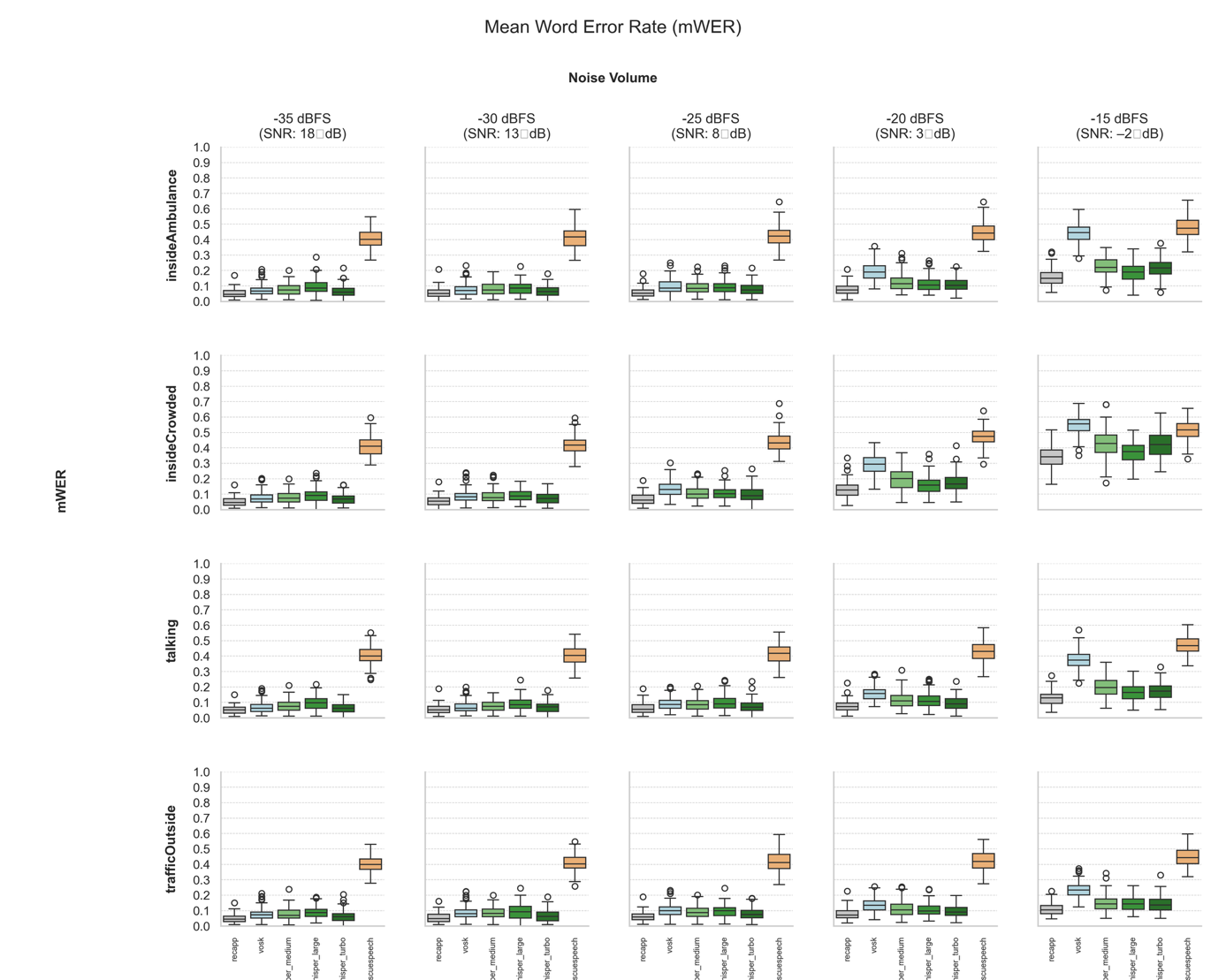


Figure S3. Boxplots of TF-IDF cosine similarity for six STT models across four noise types (rows) and five noise volume levels (columns); n = 99 per box (total n = 11880).


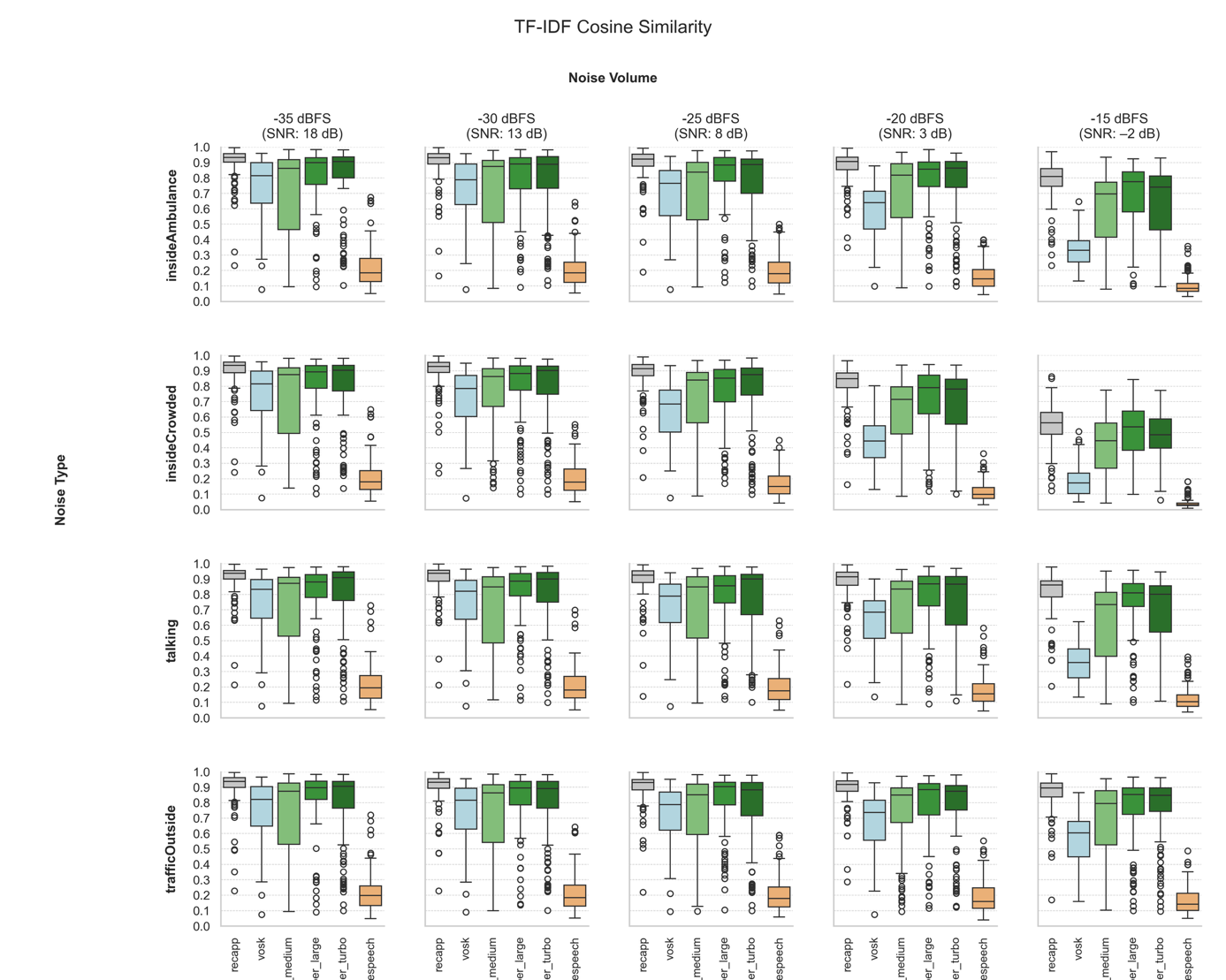


Figure S4. Boxplots of BLEU for six STT models across four noise types (rows) and five noise volume levels (columns); n = 99 per box (total n = 11880). The RescueSpeech model achieves BLEU scores near zero and therefore its boxes are not visible at this scale.


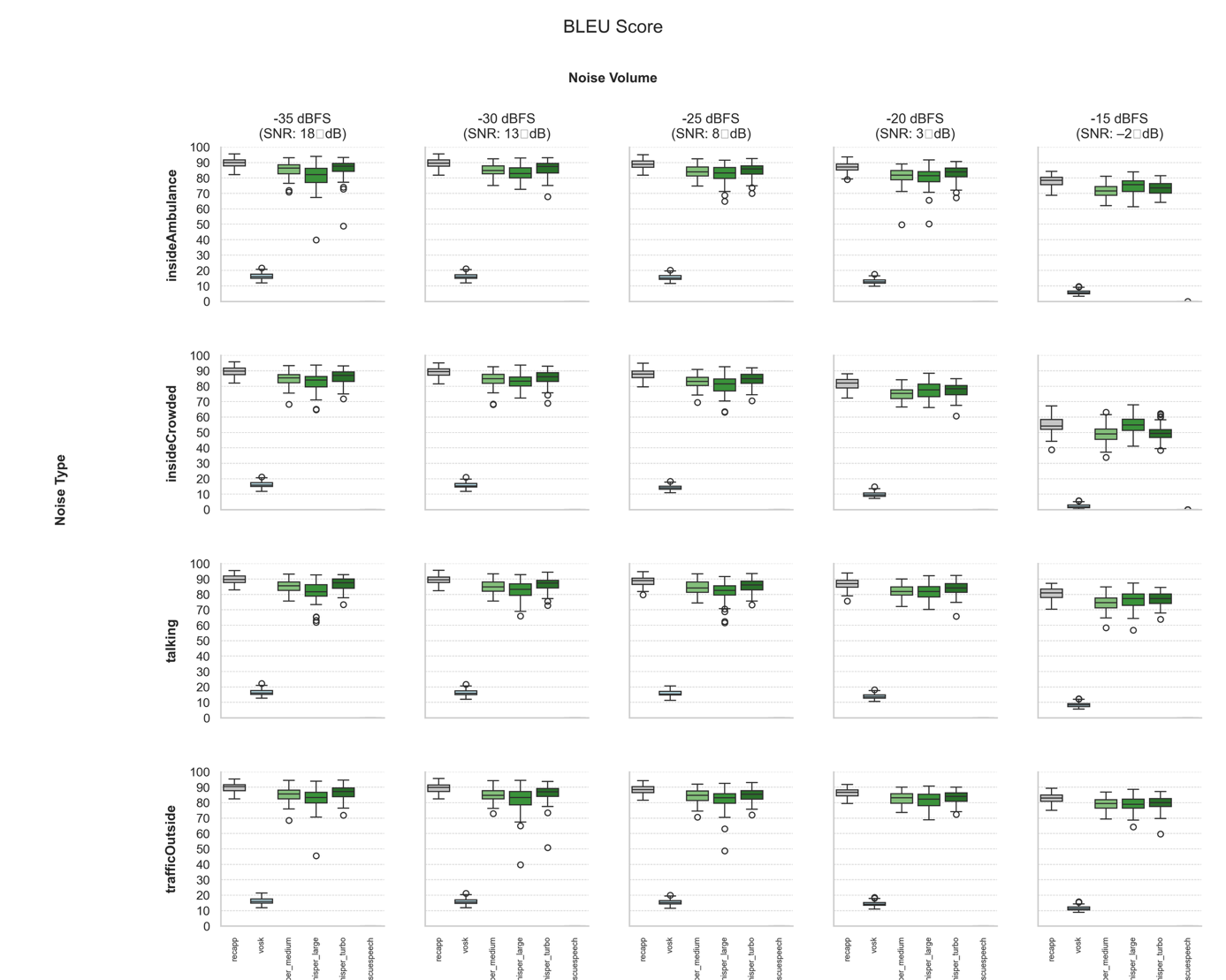


Figure S5. Boxplots of semantic cosine similarity for six STT models across four noise types (rows) and five noise volume levels (columns); n = 99 per box (total n = 11880).


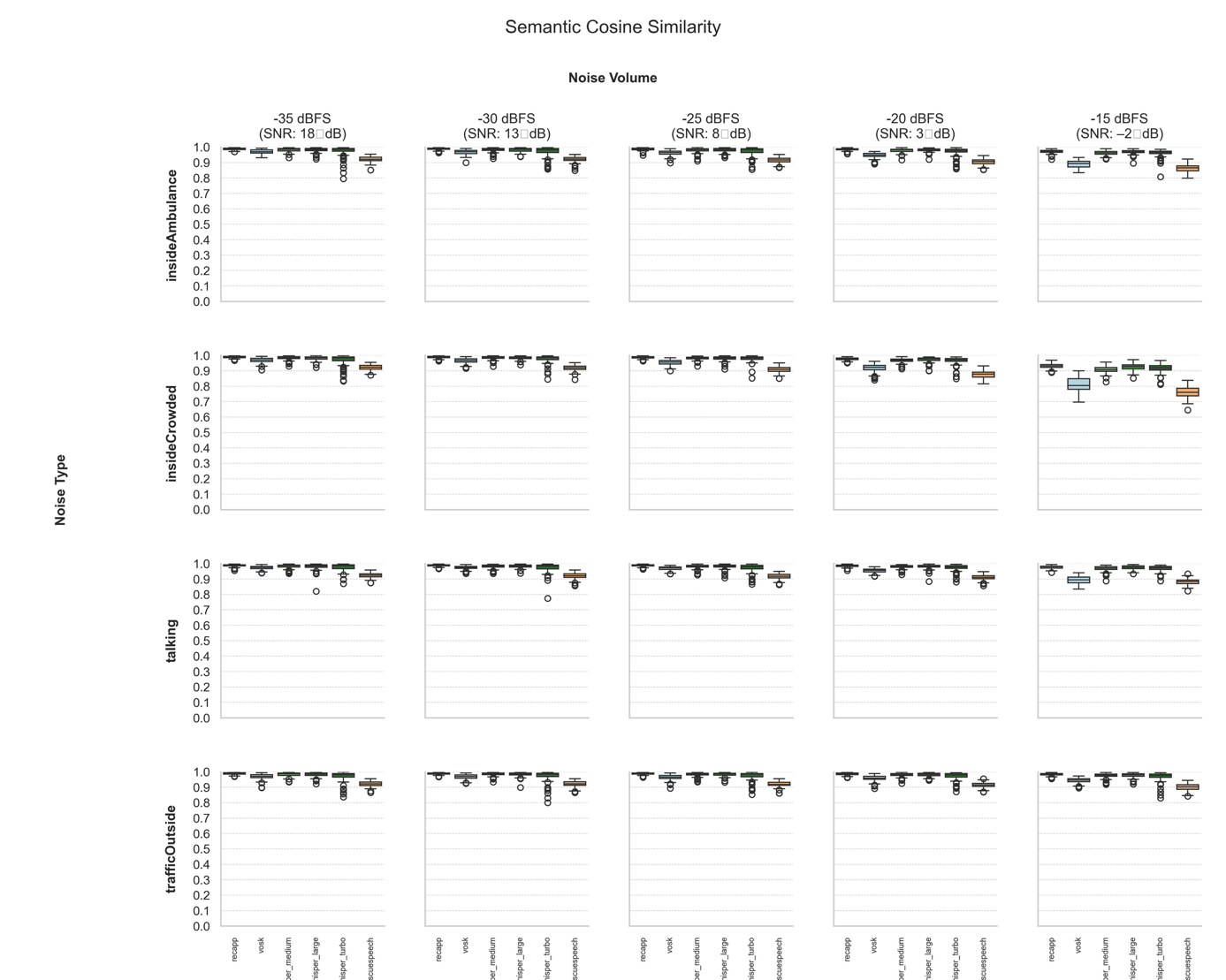


Figure S6. SHAP beeswarm plot illustrating feature importance and impact on predicted medical Word Error Rate (mWER) in the gradient boosting model. Features are ranked by importance (top = most important); colors indicate feature value (red = high, blue = low).


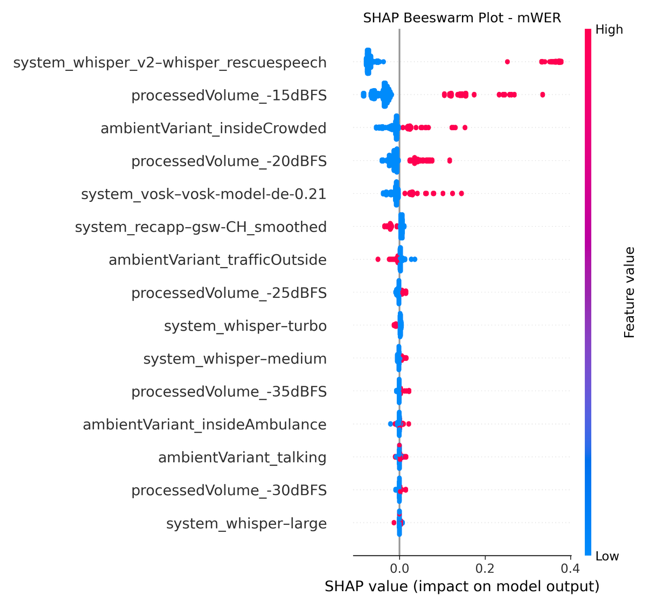


Figure S7. Feature importance ranking (mean absolute SHAP values) for predicting medical Word Error Rate (mWER) using gradient boosting. Higher mean SHAP values indicate greater average impact on the model's predictions.


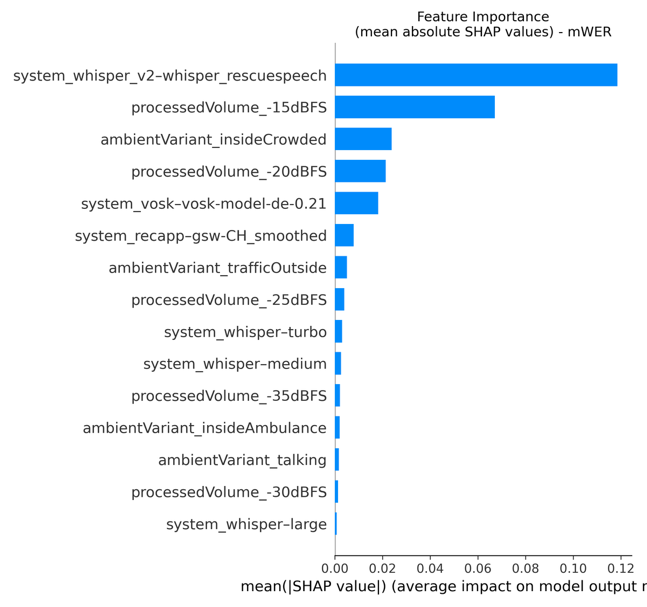

Supplement: ooaf147_Supplementary_Data [file ooaf147_supplementary_data.zip › Supplementary-Figures-JAMIA.docx]
