# Supplementary material for: Benchmarking speech-to-text robustness in noisy emergency medical dialogues: an evaluation of models under realistic acoustic conditions
Source: JAMIA Open. 2025 Nov 19;8(6):ooaf147. doi: 10.1093/jamiaopen/ooaf147 (PMC12628192; doi:10.1093/jamiaopen/ooaf147)
Supplement: ooaf147_Supplementary_Data [file ooaf147_supplementary_data.zip › Supplementary-Tables-JAMIA.docx]

# Supplementary Tables

Table S1. Descriptive statistics of word error rate (WER) across STT models (n = 1980 per model; total n = 11880; 99 utterances × 4 noise types × 5 volume levels).

| **Descriptive Statistics - WER** | | | | | | |  |
| --- | --- | --- | --- | --- | --- | --- | --- |
| **model** | **technology** | **mean** | **median** | **std** | **min** | **max** | |
| gsw-CH_smoothed | recapp | 0.083 | 0.061 | 0.074 | 0.015 | 0.537 | |
| large | vosk | 0.239 | 0.161 | 0.182 | 0.043 | 0.852 | |
| medium | whisper | 0.157 | 0.143 | 0.084 | 0.022 | 0.593 | |
| turbo | whisper | 0.122 | 0.101 | 0.085 | 0.021 | 0.674 | |
| vosk-model-de-0.21 | whisper | 0.108 | 0.083 | 0.089 | 0.023 | 0.655 | |
| whisper_rescuespeech | whisper_v2 | 0.601 | 0.580 | 0.099 | 0.429 | 0.921 | |

Table S2. Variance inflation factors (VIF) for predictors in the OLS model of word error rate (WER).

| **WER - Variance Inflation Factor** | |
| --- | --- |
| **variable** | **VIF** |
| Intercept | 13.000 |
| C(system)[T.whisper–large] | 1.667 |
| C(system)[T.whisper–medium] | 1.667 |
| C(system)[T.whisper–turbo] | 1.667 |
| C(system)[T.vosk–vosk-model-de-0.21] | 1.667 |
| C(system)[T.whisper_v2–whisper_rescuespeech] | 1.667 |
| C(ambientVariant)[T.insideAmbulance] | 1.500 |
| C(ambientVariant)[T.insideCrowded] | 1.500 |
| C(ambientVariant)[T.talking] | 1.500 |
| C(processedVolume)[T.-15dBFS] | 1.600 |
| C(processedVolume)[T.-20dBFS] | 1.600 |
| C(processedVolume)[T.-25dBFS] | 1.600 |
| C(processedVolume)[T.-30dBFS] | 1.600 |

Table S3. Ordinary least squares (OLS) regression predicting word error rate (WER). Baseline categories (reference groups) are: **STT model** = recapp^TM^, **noise type** = trafficOutside, and **noise volume** = –35 dBFS.

| **WER - OLS Regression (cluster-robust SE)** | | | | | | |
| --- | --- | --- | --- | --- | --- | --- |
|  | **Coef.** | **Std.Err.** | **t** | **P>\|t\|** | **[0.025** | **0.975]** |
| Intercept | 0.001 | 0.002 | 0.312 | 0.756 | -0.003 | 0.005 |
| C(system)[T.whisper–large] | 0.075 | 0.002 | 37.279 | 0.000 | 0.071 | 0.079 |
| C(system)[T.whisper–medium] | 0.039 | 0.001 | 26.851 | 0.000 | 0.036 | 0.042 |
| C(system)[T.whisper–turbo] | 0.026 | 0.002 | 16.120 | 0.000 | 0.023 | 0.029 |
| C(system)[T.vosk–vosk-model-de-0.21] | 0.156 | 0.002 | 71.616 | 0.000 | 0.152 | 0.161 |
| C(system)[T.whisper_v2–whisper_rescuespeech] | 0.518 | 0.004 | 135.286 | 0.000 | 0.511 | 0.526 |
| C(ambientVariant)[T.insideAmbulance] | 0.019 | 0.001 | 23.977 | 0.000 | 0.017 | 0.020 |
| C(ambientVariant)[T.insideCrowded] | 0.079 | 0.001 | 58.020 | 0.000 | 0.076 | 0.081 |
| C(ambientVariant)[T.talking] | 0.007 | 0.001 | 9.358 | 0.000 | 0.005 | 0.008 |
| C(processedVolume)[T.-15dBFS] | 0.196 | 0.003 | 76.599 | 0.000 | 0.191 | 0.201 |
| C(processedVolume)[T.-20dBFS] | 0.062 | 0.001 | 48.142 | 0.000 | 0.059 | 0.064 |
| C(processedVolume)[T.-25dBFS] | 0.018 | 0.001 | 21.852 | 0.000 | 0.016 | 0.019 |
| C(processedVolume)[T.-30dBFS] | 0.004 | 0.001 | 5.454 | 0.000 | 0.002 | 0.005 |

Table S4. Descriptive statistics of medical word error rate (mWER) across STT models (n = 1980 per model; total n = 11880; 99 utterances × 4 noise types × 5 volume levels).

| **mWER - Descriptive Statistics** | | | | | | |
| --- | --- | --- | --- | --- | --- | --- |
| **model** | **technology** | **mean** | **median** | **std** | **min** | **max** |
| gsw-CH_smoothed | recapp | 0.088 | 0.068 | 0.076 | 0.000 | 0.517 |
| large | whisper | 0.124 | 0.106 | 0.080 | 0.000 | 0.515 |
| medium | whisper | 0.128 | 0.103 | 0.096 | 0.007 | 0.682 |
| turbo | whisper | 0.114 | 0.088 | 0.094 | 0.000 | 0.626 |
| vosk-model-de-0.21 | vosk | 0.171 | 0.115 | 0.143 | 0.000 | 0.688 |
| whisper_rescuespeech | whisper_v2 | 0.433 | 0.429 | 0.070 | 0.247 | 0.688 |

TableS5. Variance inflation factors (VIF) for predictors in the OLS model of medical word error rate (mWER).

| **mWER - Variance Inflation Factor** | |
| --- | --- |
| **variable** | **VIF** |
| system_whisper–large | 0.001 |
| system_whisper–medium | 0.001 |
| system_whisper–turbo | 0.001 |
| system_vosk–vosk-model-de-0.21 | 0.001 |
| system_whisper_v2–whisper_rescuespeech | 0.001 |
| ambientVariant_insideAmbulance | 0.001 |
| ambientVariant_insideCrowded | 0.001 |
| ambientVariant_talking | 0.001 |
| processedVolume_-15dBFS | 0.001 |
| processedVolume_-20dBFS | 0.001 |
| processedVolume_-25dBFS | 0.001 |
| processedVolume_-30dBFS | 0.001 |

Table S6. Ordinary least squares (OLS) regression predicting medical word error rate (mWER). Baseline categories (reference groups) are: **STT model** = recapp^TM^, **noise type** = trafficOutside, and **noise volume** = –35 dBFS.

| **mWER -** **OLS Regression (cluster-robust SE)** | | | | | | | |
| --- | --- | --- | --- | --- | --- | --- | --- |
|  | **Coef.** | **Std.Err.** | **t** | **P>\|t\|** | **[0.025** | **0.975]** |  |
| Intercept | 0.016 | 0.003 | 5.436 | 0.000 | 0.010 | 0.022 |  |
| C(system)[T.whisper–large] | 0.035 | 0.001 | 26.871 | 0.000 | 0.033 | 0.038 |  |
| C(system)[T.whisper–medium] | 0.040 | 0.002 | 19.135 | 0.000 | 0.036 | 0.044 |  |
| C(system)[T.whisper–turbo] | 0.026 | 0.002 | 15.782 | 0.000 | 0.023 | 0.029 |  |
| C(system)[T.vosk–vosk-model-de-0.21] | 0.083 | 0.003 | 27.506 | 0.000 | 0.077 | 0.089 |  |
| C(system)[T.whisper_v2–whisper_rescuespeech] | 0.344 | 0.006 | 61.124 | 0.000 | 0.333 | 0.355 |  |
| C(ambientVariant)[T.insideAmbulance] | 0.019 | 0.001 | 18.002 | 0.000 | 0.017 | 0.021 |  |
| C(ambientVariant)[T.insideCrowded] | 0.067 | 0.002 | 42.419 | 0.000 | 0.064 | 0.070 |  |
| C(ambientVariant)[T.talking] | 0.009 | 0.001 | 8.611 | 0.000 | 0.007 | 0.011 |  |
| C(processedVolume)[T.-15dBFS] | 0.167 | 0.003 | 59.804 | 0.000 | 0.161 | 0.172 |  |
| C(processedVolume)[T.-20dBFS] | 0.057 | 0.002 | 33.334 | 0.000 | 0.053 | 0.060 |  |
| C(processedVolume)[T.-25dBFS] | 0.017 | 0.001 | 16.923 | 0.000 | 0.015 | 0.019 |  |
| C(processedVolume)[T.-30dBFS] | 0.004 | 0.001 | 5.956 | 0.000 | 0.003 | 0.005 |  |

Table S7. Descriptive statistics of TD-IDF cosine similarity across STT models (n = 1980 per model; total n = 11880; 99 utterances × 4 noise types × 5 volume levels).

| **TD-IDF Cosine Similarity - Descriptive Statistics** | | | | | | |
| --- | --- | --- | --- | --- | --- | --- |
| **model** | **technology** | **mean** | **median** | **std** | **min** | **max** |
| gsw-CH_smoothed | recapp | 0.856 | 0.911 | 0.148 | 0.122 | 0.997 |
| large | whisper | 0.766 | 0.856 | 0.219 | 0.091 | 0.984 |
| medium | whisper | 0.695 | 0.817 | 0.260 | 0.041 | 0.987 |
| turbo | whisper | 0.741 | 0.862 | 0.250 | 0.061 | 0.983 |
| vosk-model-de-0.21 | vosk | 0.627 | 0.684 | 0.239 | 0.050 | 0.965 |
| whisper_rescuespeech | whisper_v2 | 0.180 | 0.148 | 0.115 | 0.008 | 0.729 |

Table S8. Variance inflation factors (VIF) for predictors in the OLS model of TD-IDF cosine similarity.

| **TF-IDF Cosine Similarity - Variance Inflation Factor** | |
| --- | --- |
| **variable** | **VIF** |
| Intercept | 13.000 |
| C(system)[T.whisper–large] | 1.667 |
| C(system)[T.whisper–medium] | 1.667 |
| C(system)[T.whisper–turbo] | 1.667 |
| C(system)[T.vosk–vosk-model-de-0.21] | 1.667 |
| C(system)[T.whisper_v2–whisper_rescuespeech] | 1.667 |
| C(ambientVariant)[T.insideAmbulance] | 1.500 |
| C(ambientVariant)[T.insideCrowded] | 1.500 |
| C(ambientVariant)[T.talking] | 1.500 |
| C(processedVolume)[T.-15dBFS] | 1.600 |
| C(processedVolume)[T.-20dBFS] | 1.600 |
| C(processedVolume)[T.-25dBFS] | 1.600 |
| C(processedVolume)[T.-30dBFS] | 1.600 |

Table S9. Ordinary least squares (OLS) regression predicting TD-IDF cosine similarity. Baseline categories (reference groups) are: **STT model** = recapp^TM^, **noise type** = trafficOutside, and **noise volume** = –35 dBFS.

| **TF-IDF Cosine Similarity - OLS Regression (cluster-robust SE)** | | | | | | |
| --- | --- | --- | --- | --- | --- | --- |
|  | **Coef.** | **Std.Err.** | **t** | **P>\|t\|** | **[0.025** | **0.975]** |
| Intercept | 0.946 | 0.013 | 70.892 | 0.000 | 0.919 | 0.972 |
| C(system)[T.whisper–large] | -0.091 | 0.009 | -10.246 | 0.000 | -0.108 | -0.073 |
| C(system)[T.whisper–medium] | -0.161 | 0.017 | -9.266 | 0.000 | -0.196 | -0.127 |
| C(system)[T.whisper–turbo] | -0.116 | 0.014 | -8.207 | 0.000 | -0.144 | -0.088 |
| C(system)[T.vosk–vosk-model-de-0.21] | -0.230 | 0.016 | -14.586 | 0.000 | -0.261 | -0.198 |
| C(system)[T.whisper_v2–whisper_rescuespeech] | -0.676 | 0.016 | -43.357 | 0.000 | -0.707 | -0.645 |
| C(ambientVariant)[T.insideAmbulance] | -0.030 | 0.002 | -13.666 | 0.000 | -0.035 | -0.026 |
| C(ambientVariant)[T.insideCrowded] | -0.084 | 0.004 | -20.524 | 0.000 | -0.093 | -0.076 |
| C(ambientVariant)[T.talking] | -0.022 | 0.003 | -7.539 | 0.000 | -0.028 | -0.016 |
| C(processedVolume)[T.-15dBFS] | -0.178 | 0.006 | -31.080 | 0.000 | -0.190 | -0.167 |
| C(processedVolume)[T.-20dBFS] | -0.066 | 0.004 | -15.494 | 0.000 | -0.074 | -0.057 |
| C(processedVolume)[T.-25dBFS] | -0.024 | 0.003 | -7.859 | 0.000 | -0.030 | -0.018 |
| C(processedVolume)[T.-30dBFS] | -0.007 | 0.002 | -2.896 | 0.005 | -0.011 | -0.002 |

Table S10. Descriptive statistics of BLEU Scores across STT models (n = 1980 per model; total n = 11880; 99 utterances × 4 noise types × 5 volume levels)

| **BLEU - Descriptive Statistics** | | | | | | |
| --- | --- | --- | --- | --- | --- | --- |
| **model** | **technology** | **mean** | **median** | **std** | **min** | **max** |
| gsw-CH_smoothed | recapp | 85.283 | 87.306 | 8.344 | 38.893 | 95.823 |
| large | whisper | 79.769 | 81.180 | 8.292 | 39.740 | 94.443 |
| medium | whisper | 80.492 | 82.515 | 9.200 | 33.921 | 94.483 |
| turbo | whisper | 81.827 | 84.028 | 9.321 | 38.518 | 94.635 |
| vosk-model-de-0.21 | vosk | 13.465 | 14.468 | 4.252 | 0.294 | 22.186 |
| whisper_rescuespeech | whisper_v2 | 0.010 | 0.008 | 0.005 | 0.000 | 0.027 |

Table S11. Variance inflation factors (VIF) for predictors in the OLS model of BLEU

| **BLEU - Variance Inflation Factor** | |
| --- | --- |
| **variable** | **VIF** |
| Intercept | 13.000 |
| C(system)[T.whisper–large] | 1.667 |
| C(system)[T.whisper–medium] | 1.667 |
| C(system)[T.whisper–turbo] | 1.667 |
| C(system)[T.vosk–vosk-model-de-0.21] | 1.667 |
| C(system)[T.whisper_v2–whisper_rescuespeech] | 1.667 |
| C(ambientVariant)[T.insideAmbulance] | 1.500 |
| C(ambientVariant)[T.insideCrowded] | 1.500 |
| C(ambientVariant)[T.talking] | 1.500 |
| C(processedVolume)[T.-15dBFS] | 1.600 |
| C(processedVolume)[T.-20dBFS] | 1.600 |
| C(processedVolume)[T.-25dBFS] | 1.600 |
| C(processedVolume)[T.-30dBFS] | 1.600 |

Table S12. Ordinary least squares (OLS) regression predicting BLEU Scores. Baseline categories (reference groups) are: **STT model** = recapp^TM^, **noise type** = trafficOutside, and **noise volume** = –35 dBFS.

| **BLEU - OLS Regression (cluster-robust SE)** | | | | | | |
| --- | --- | --- | --- | --- | --- | --- |
|  | **Coef.** | **Std.Err.** | **t** | **P>\|t\|** | **[0.025** | **0.975]** |
| Intercept | 90.048 | 0.302 | 297.939 | 0.000 | 89.448 | 90.647 |
| C(system)[T.whisper–large] | -5.514 | 0.187 | -29.515 | 0.000 | -5.885 | -5.143 |
| C(system)[T.whisper–medium] | -4.791 | 0.203 | -23.583 | 0.000 | -5.194 | -4.388 |
| C(system)[T.whisper–turbo] | -3.456 | 0.222 | -15.562 | 0.000 | -3.897 | -3.016 |
| C(system)[T.vosk–vosk-model-de-0.21] | -71.818 | 0.309 | -232.566 | 0.000 | -72.431 | -71.205 |
| C(system)[T.whisper_v2–whisper_rescuespeech] | -85.273 | 0.282 | -302.472 | 0.000 | -85.832 | -84.713 |
| C(ambientVariant)[T.insideAmbulance] | -1.016 | 0.071 | -14.259 | 0.000 | -1.157 | -0.874 |
| C(ambientVariant)[T.insideCrowded] | -5.134 | 0.101 | -50.754 | 0.000 | -5.334 | -4.933 |
| C(ambientVariant)[T.talking] | -0.408 | 0.071 | -5.744 | 0.000 | -0.549 | -0.267 |
| C(processedVolume)[T.-15dBFS] | -11.471 | 0.180 | -63.809 | 0.000 | -11.828 | -11.114 |
| C(processedVolume)[T.-20dBFS] | -3.171 | 0.097 | -32.594 | 0.000 | -3.364 | -2.978 |
| C(processedVolume)[T.-25dBFS] | -0.864 | 0.074 | -11.740 | 0.000 | -1.010 | -0.718 |
| C(processedVolume)[T.-30dBFS] | -0.120 | 0.059 | -2.044 | 0.044 | -0.237 | -0.004 |

Table S13. Descriptive statistics of semantic cosine similarity across STT models (n = 1980 per model; total n = 11880; 99 utterances × 4 noise types × 5 volume levels).

| **Semantic Cosine Similarity - Descriptive Statistics** | | | | | | |
| --- | --- | --- | --- | --- | --- | --- |
| **model** | **technology** | **mean** | **median** | **std** | **min** | **max** |
| gsw-CH_smoothed | recapp | 0.982 | 0.987 | 0.015 | 0.890 | 0.998 |
| large | whisper | 0.977 | 0.983 | 0.019 | 0.820 | 0.998 |
| medium | whisper | 0.975 | 0.982 | 0.021 | 0.828 | 0.998 |
| turbo | whisper | 0.969 | 0.979 | 0.030 | 0.775 | 0.998 |
| vosk-model-de-0.21 | vosk | 0.946 | 0.960 | 0.044 | 0.697 | 0.994 |
| whisper_rescuespeech | whisper_v2 | 0.902 | 0.912 | 0.042 | 0.646 | 0.959 |

Table S14. Variance inflation factors (VIF) for predictors in the OLS model of semantic cosine similarity.

| **Semantic Cosine Similarity - Variance Inflation Factor** | |
| --- | --- |
| **variable** | **VIF** |
| system_whisper–large | 0.001 |
| system_whisper–medium | 0.001 |
| system_whisper–turbo | 0.001 |
| system_vosk–vosk-model-de-0.21 | 0.001 |
| system_whisper_v2–whisper_rescuespeech | 0.001 |
| ambientVariant_insideAmbulance | 0.001 |
| ambientVariant_insideCrowded | 0.001 |
| ambientVariant_talking | 0.001 |
| processedVolume_-15dBFS | 0.001 |
| processedVolume_-20dBFS | 0.001 |
| processedVolume_-25dBFS | 0.001 |
| processedVolume_-30dBFS | 0.001 |

Table S15. Ordinary least squares (OLS) regression predicting semantic cosine similarity. Baseline categories (reference groups) are: **STT model** = recapp^TM^, **noise type** = trafficOutside, and **noise volume** = –35 dBFS.

| **Semantic Cosine Similarity – OLS Regression (cluster-robust SE)** | | | | | | |
| --- | --- | --- | --- | --- | --- | --- |
|  | **Coef.** | **Std.Err.** | **t** | **P>\|t\|** | **[0.025** | **0.975]** |
| Intercept | 1.001 | 0.001 | 1133.017 | 0.000 | 0.999 | 1.003 |
| C(system)[T.whisper–large] | -0.005 | 0.000 | -12.411 | 0.000 | -0.006 | -0.005 |
| C(system)[T.whisper–medium] | -0.007 | 0.001 | -10.488 | 0.000 | -0.009 | -0.006 |
| C(system)[T.whisper–turbo] | -0.014 | 0.001 | -13.370 | 0.000 | -0.016 | -0.012 |
| C(system)[T.vosk–vosk-model-de-0.21] | -0.037 | 0.001 | -34.572 | 0.000 | -0.039 | -0.035 |
| C(system)[T.whisper_v2–whisper_rescuespeech] | -0.080 | 0.002 | -53.430 | 0.000 | -0.083 | -0.077 |
| C(ambientVariant)[T.insideAmbulance] | -0.006 | 0.000 | -13.917 | 0.000 | -0.006 | -0.005 |
| C(ambientVariant)[T.insideCrowded] | -0.021 | 0.001 | -30.557 | 0.000 | -0.023 | -0.020 |
| C(ambientVariant)[T.talking] | -0.003 | 0.000 | -6.644 | 0.000 | -0.003 | -0.002 |
| C(processedVolume)[T.-15dBFS] | -0.041 | 0.001 | -42.125 | 0.000 | -0.043 | -0.039 |
| C(processedVolume)[T.-20dBFS] | -0.011 | 0.000 | -25.166 | 0.000 | -0.011 | -0.010 |
| C(processedVolume)[T.-25dBFS] | -0.003 | 0.000 | -7.357 | 0.000 | -0.004 | -0.002 |
| C(processedVolume)[T.-30dBFS] | 0.000 | 0.000 | -1.274 | 0.206 | -0.001 | 0.000 |

Table S16. Mixed-effects linear regression predicting medical Word Error Rate (mWER). Baseline categories (reference groups) are: **STT model** = recapp^TM^, **noise type** = trafficOutside, and **noise volume** = –35 dBFS.

| **mWER – Mixed-Effects Linear Model Regression Results** | | | | | | |
| --- | --- | --- | --- | --- | --- | --- |
| **variable** | **Coef.** | **Std.Err.** | **z** | **P>\|z\|** | **[0.025** | **0.975]** |
| Intercept | 0.016 | 0.003 | 4.779 | 0.000 | 0.009 | 0.023 |
| C(system)[T.whisper–large] | 0.035 | 0.002 | 17.542 | 0.000 | 0.031 | 0.039 |
| C(system)[T.whisper–medium] | 0.040 | 0.002 | 19.725 | 0.000 | 0.036 | 0.044 |
| C(system)[T.whisper–turbo] | 0.026 | 0.002 | 12.868 | 0.000 | 0.022 | 0.030 |
| C(system)[T.vosk–vosk-model-de-0.21] | 0.083 | 0.002 | 41.018 | 0.000 | 0.079 | 0.087 |
| C(system)[T.whisper_v2–whisper_rescuespeech] | 0.344 | 0.002 | 170.653 | 0.000 | 0.340 | 0.348 |
| C(ambientVariant)[T.insideAmbulance] | 0.019 | 0.002 | 11.514 | 0.000 | 0.016 | 0.022 |
| C(ambientVariant)[T.insideCrowded] | 0.067 | 0.002 | 40.679 | 0.000 | 0.064 | 0.070 |
| C(ambientVariant)[T.talking] | 0.009 | 0.002 | 5.267 | 0.000 | 0.005 | 0.012 |
| C(processedVolume)[T.-15dBFS] | 0.167 | 0.002 | 90.676 | 0.000 | 0.163 | 0.171 |
| C(processedVolume)[T.-20dBFS] | 0.057 | 0.002 | 30.708 | 0.000 | 0.053 | 0.060 |
| C(processedVolume)[T.-25dBFS] | 0.017 | 0.002 | 9.027 | 0.000 | 0.013 | 0.020 |
| C(processedVolume)[T.-30dBFS] | 0.004 | 0.002 | 2.169 | 0.030 | 0.000 | 0.008 |
| Group Var | 0.001 | 0.002 |  |  |  |  |

Table S17. Selected transcription examples highlighting typical STT errors such as substitutions and hallucinations, notably in RescueSpeech.

| Reference Text | *“ Frau Meier, wir werden Ihnen jetzt ein Schmerzmittel geben, um Ihre Beschwerden zu lindern. Es enthält Acetaminophen und Codein.”* |
| --- | --- |
| Hypothesis – Whisper V3 Turbo | *„Frau Meier, wir werden Ihnen jetzt ein Schmerzmittel geben, um Ihre Beschwerden zu lindern. Es enthält Acetaminophen und Coten.“* |
| Hypothesis –  Vosk | *„frau meyer wir werden ihnen jetzt ein schmerzmittel geben um ihre beschwerden zu lindern es enthält acetaminophen und koten“* |
| Hypothesis –  RescueSpeech | *„FRAU MEIR WIR WERDEN IHNEN JETZT AN SCHMEITEL GEBEN UM IHRE BESCHWERDEN ZU LINDERN ES ENTHÄLT AKITAMINOFEN UND KOTEN”* |
